# Assessment of Electronic Clinical Monitoring Systems in the Pediatric Intensive Care Unit: Prospective Concordance Study

**DOI:** 10.2196/93017

**Published:** 2026-08-11

**Authors:** David Pham, Patricia Tachinardi, Lucas Bulgarelli, Wellington Dos Reis Lucena, Eneida Mendonca, Colin Rogerson

**Affiliations:** 1 Department of Pediatrics, Indiana University School of Medicine, 635 Barnhill Drive, Rm 112, Indianapolis, IN, 46202, United States, 1 317-274-3772; 2 Department of Pediatrics, Cincinnati Children's Hospital Medical Center, Cincinnati, OH, United States

**Keywords:** pediatrics, critical care, informatics, electronic health records, clinical research

## Abstract

**Background:**

Electronic health record (EHR) data are being increasingly used for retrospective observational research through large, robust databases, and advanced data extraction tools.

**Objective:**

We sought to assess the reliability of vital sign, ventilator, and continuous medication data captured in the EHR in a pediatric intensive care unit.

**Methods:**

We conducted a prospective concordance study of children receiving invasive mechanical ventilation in June 2025. Data sources included (1) a bedside clinical researcher, (2) automated EHR extraction, and (3) a continuous vital sign monitoring system. Vital signs from the EHR were compared to those obtained through the continuous vital sign monitoring system. Ventilator and medication data were compared to the bedside observations. Differences were measured as means with SDs or median differences with IQRs, and a 10% error rate was used as a concordance adequacy threshold.

**Results:**

We obtained 110 bedside observations from 27 unique patients. Five of 8 measured vital signs in the EHR met the 10% concordance adequacy threshold (respiratory rate, 2.0/20.4, 9.8%; cuff systolic blood pressure, 8.3/99.4, 8.4%; invasive systolic blood pressure, 4.0/88.0, 4.5%; invasive diastolic blood pressure, 3.8/50.0, 7.6%; and oxygen saturation, 2/96, 2.1%), with heart rate (11.4/113, 10.1%), cuff diastolic blood pressure (9/58.6, 15.3%), and end-tidal carbon dioxide (4.0/36.6, 10.9%) failing to meet concordance. Occasional rare clinically meaningful outliers were observed, such as a systolic blood pressure difference of 31 mm Hg, a heart rate difference of 87 beats per minute, and a respiratory rate difference of 18 breaths per minute. All 6 ventilator settings met concordance adequacy criteria between the EHR and the bedside observations, with a median difference of 0.0 (IQR 0-0). Outliers were less common but included differences such as a respiratory rate of 34 breaths per minute and an inspiratory time of 0.3 seconds. Continuous medication dosing concordance was variable, with an overall low concordance between 30.8% (339.3/11) and 31.4% (345.4/11).

**Conclusions:**

EHR data captured in the pediatric intensive care unit in our single-center sample were mostly concordant with other measured observations for vital signs and ventilator settings, but less concordant for continuous medications.

## Introduction

The electronic health record (EHR) has become the standard for clinical documentation in the United States [1,2]. EHR systems enable hospitals to collect and store vast amounts of patient data in near real time, mostly through manual input from bedside nursing staff [3,4]. While the primary purpose of these data is patient care, a valuable secondary use is observational research [5-8]. Clinical data collection in the intensive care unit (ICU) is unique and facilitates rapid recognition of clinical status changes that support immediate and life-saving decisions. These advanced monitoring systems include data regarding vital signs, medications, ventilator settings, etc [9,10]. While the scope of these data provides many opportunities for observational clinical research, the volume and variety of data raise concerns for potential data quality issues [11-13].

Although EHR data are assumed to be accurate and often used in observational research, this assumption is largely unproven. Prior studies have highlighted the need for further research on EHR data quality, including completeness, plausibility, currency, and correctness and accuracy [14-17]. There is evidence that EHR vital sign data accuracy is dependent on factors such as hospital settings, specialty, and patient demographics [18]. Furthermore, data accuracy is also impacted by challenges to the documentation workflow, such as interface complexity of EHR systems, workflow fragmentation, documentation duplication (eg, handwriting notes at the bedside and later entering the data into the EHR system), and misalignment with clinical workflows [19]. In the ICU, data accuracy may vary greatly between the different types of data recorded and different data capture methods [20,21].

While most EHR data are obtained by bedside nursing staff and manually entered into the EHR system, novel informatics tools are being developed to autonomously extract data elements continuously recorded by bedside monitors. In a previous study in a pediatric cardiac ICU setting, it was shown that vital sign documentation is incomplete in conventional EHR charting when compared to data aggregated from an automated platform, which led to substantial underrepresentation of acute vital sign changes in the conventional EHR dataset [22]. The objective of this study was to compare the concordance of captured data in the EHR vs data captured in real time using either a continuous vital sign monitoring system or bedside data collection among intubated patients in the pediatric ICU (PICU). Specifically, our goal was to determine the rate and magnitude of discordance between data manually recorded at the bedside by a clinical research team member, data recorded in the EHR during the routine process of patient care by bedside health care professionals, and data captured via an automated platform. We also analyzed the discordance between different categories of data (ie, vital sign data vs ventilator data vs medication data). We hypothesized that data captured in the EHR would be discordant with data captured through the other stated methods.

## Methods

### Study Population

We conducted a prospective concordance study at a single, quaternary care academic pediatric institution. The study population included all patients admitted to the Riley Hospital for Children PICU receiving invasive mechanical ventilation during the study period. Invasive ventilation included both via endotracheal tube or tracheostomy. We chose this population to maximize the study data in terms of both breadth and depth as these patients will have regular data collected on vital signs, ventilator settings, and continuous medications. Children at the end of life or with significant social concerns that could be exacerbated by the research team member’s presence were excluded. The study period was June 4 to 27, 2025. This study was reported using the STROBE (Strengthening the Reporting of Observational Studies in Epidemiology) guidelines (Multimedia Appendix 1) [23].

### Ethical Considerations

This study was reviewed by the Indiana University Institutional Review Board (27592) and approved after expedited review with a waiver of informed consent due to the minimal-risk observational design. Research personnel observed and recorded bedside data without direct interaction with patients or involvement in clinical care. This study was conducted in accordance with the ethical principles of the Declaration of Helsinki.

### Data Collection and Elements

Data were collected using 3 different methods. The first method was data collection at the bedside by a trained clinical research team member. The research team member was present in the PICU on various dates over the study period and at various times of day between 7 AM and 7 PM, with one collection per day. The research team member documented real-time data into a secure Indiana University Google Drive while at the bedside and conducted 18 distinct collections over the study period. Each collection event was treated as a distinct episode, and the same patient could have multiple episodes of collection over multiple days. Bedside data were collected using a standardized worksheet and protocol each time. Data obtained through the bedside worksheet were evaluated for documentation accuracy and outliers 3 times over the study period. The second method of data capture was via automated extraction from the EHR system, which at our institution is a Cerner enterprise data warehouse. The standard practice in our PICU is for patients to have vital signs measured every 1 to 2 hours depending on clinical status, and respiratory therapists document ventilator settings every 4 hours. EHR vital signs are recorded by bedside nurses using the in-room patient monitors. The third method was via extraction of continuous monitoring data from the Etiometry system. Etiometry captures data from connected bedside machinery in real time, including percutaneous pulse oximeters, chest electrocardiogram leads connected to Phillips monitors, and mechanical ventilators. It then stores vital signs recorded as frequently as every 5 seconds and stores them on a secure server. Time is synchronized between the Etiometry system and the Cerner system via the time recorded on the Phillips bedside monitor. The bedside research staff used the time displayed on the bedside monitor for all recorded observations.

Data elements captured varied depending on the method of capture (Table S1 in Multimedia Appendix 1). For the data collected at the bedside, the research team member recorded vital signs (heart rate [HR], respiratory rate [RR], systolic blood pressure [SBP], diastolic blood pressure [DBP], oxygen saturation [SpO_2_], and end-tidal carbon dioxide [EtCO_2_]) upon entry into the patient room based on the most recent data recorded on the bedside monitor. For patients with an invasive arterial monitor, SBP and DBP were recorded from both the blood pressure cuff and the invasive monitor. Ventilator data were collected from the bedside ventilator and included mode, pressure control or tidal volume depending on the ventilator mode, positive end-expiratory pressure, fraction of inspired oxygen, and inspiratory time. RR was collected from both the bedside monitor and the ventilator. Medication data were collected for continuously running medications, which included sedatives (morphine, hydromorphone, fentanyl, midazolam, dexmedetomidine, and propofol), vasoactives (epinephrine, norepinephrine, dopamine, milrinone, and vasopressin), and heparin. EHR data were extracted for all the above-mentioned elements and matched to a corresponding comparison measurement. Temporal matching could occur in either direction, whichever was closest in time. For vital signs, we extracted the vital signs charted in the EHR closest to the time of the bedside recording by the research team member and matched the corresponding Etiometry values that were closest in time up to a maximum of 1 minute. For ventilator data, the ventilator settings were obtained from the research team member at the bedside via direct observation of the mechanical ventilator, and the values were compared with the EHR-charted ventilator settings closest in time to the bedside observation up to a maximum of a 12-hour difference. Medication data were obtained through direct observation of the medication infusion pumps by the research team member at the time of the bedside observation and compared to values obtained from the EHR at the time point closest to the bedside observation up to a maximum of 24 hours. Any bedside observation without a matching element obtained from the EHR or Etiometry within the maximum time limit was excluded.

Medications were recorded by the bedside researcher and from the EHR in standardized weight-based pediatric dosing, which varied between milligrams or micrograms per kilogram or milliunits per kilogram depending on the medication. Two separate medication tables were obtained from the EHR, one indicating a medication order that originated from the Cerner order table and one indicating a medication administration that originated from the medication administration record. The order table is populated by medication orders entered and removed in the EHR. The administration table is populated by an automated system as nurses use portable scanners at the bedside prior to administering medications or altering the dosing for continuous medications. Both sources were used in this study and compared to the bedside data collection. Medication errors were classified as either a dose error if the recorded infusion dose did not match the bedside infusion dose or a missing error if either the recorded medication was not actually infusing at the bedside or an infusing medication at the bedside was not recorded.

### Statistical Analysis

Comparisons were made evaluating concordance between the EHR-documented variables and other measurement methods, and varied depending on the data elements measured. Vital signs were compared between the EHR data and the continuous vital signs recorded closest in time to the EHR data time. The continuous vital sign monitoring system directly extracts data from bedside monitoring systems and has been shown in several studies to provide a more complete representation of continuously acquired physiological data than intermittent EHR documentation, which is why it was chosen as the comparison for this study [22,24-27]. Bedside vital signs collected by the research team member were not used because rapid, physiological fluctuations in vital signs rendered any comparison to EHR vital signs recorded at a different time point invalid. Ventilator settings and medication doses were compared between the extracted EHR data and the bedside recordings by the research team member. The bedside observations were used because direct bedside observation of these data is more likely to be accurate at that moment in time compared to extrapolated data entered into the EHR at time points surrounding the observation. Each bedside observation was counted as an event, the available data at that time were compared, and the difference between the measurements was calculated. Differences were summarized for each continuous variable as means with SDs for normally distributed data and medians with IQRs for non-normally distributed data. To adjust for repeated observations for the same patient, patient-level analyses were conducted. The mean absolute differences for each variable were calculated for each individual patient and then summarized across all patients. For the medication data, differences were measured in median percentage of errors with IQRs rather than median difference due to the widely variable dosing regimens across the included infusions. Median differences in percentage of error rates between the order and administration medication tables were compared using Mann-Whitney *U* tests. Discrete data were measured as frequencies with percentages and then averaged at the patient level. Comparisons of concordance between the order and administration medication tables were conducted using Wilcoxon rank-sum tests. Concordance for discrete data was determined if the ventilatory mode or medication dose in the order or administration table matched the bedside collection mode or dosing exactly. Percentage of error rates were measured for vital signs and ventilator data as the median absolute measurement error at the patient level for the cohort divided by the median measurement value for the cohort. A threshold of 10% was chosen to determine adequate concordance. Adjusted Bland-Altman plots were created for each vital sign and ventilator variable using absolute mean differences per patient (Figures S1-S14 in Multimedia Appendix 1). The R programming language (R Foundation for Statistical Computing) was used for data processing and formatting, statistical analysis, and figure creation [28]. The packages used include *dplyr*, *lubridate*, and *ggplot2*.

## Results

### Overview

Over the 23-day study period, we conducted 18 bedside data collections on 27 unique patients, resulting in 110 total bedside observations. Patients had a median age of 6 (IQR 0.9-12.5) years and a variety of primary diagnoses (Table 1). Observations per patient ranged from 1 to 18. In total, 29.6% (8/27) of the patients had invasive arterial monitoring to capture data, and all patients were connected to Etiometry continuously throughout the study period. Few observations were excluded from analysis due to inability to obtain a valid match (Table S2 in Multimedia Appendix 1).

**Table 1.** Patient characteristics (N=27).

Characteristics	Sample
Sex at birth (male), n (%)	15 (55.6)
Age (y), median (IQR)	6 (0.9-12.5)
Race, n (%)	
Black	4 (14.8)
White	22 (81.5)
Other	1 (3.7)
Ethnicity (Hispanic), n (%)	3 (11.1)
Primary diagnosis, n (%)	
Respiratory	8 (29.6)
Cardiac	6 (22.2)
Neurological	5 (18.5)
Surgical	5 (18.5)
Other	3 (11.1)

### Vital Signs

Eight vital signs were measured via EHR extraction and continuous vital sign monitoring and compared (Table 2). The vital signs with the largest median differences between the EHR data and Etiometry data were HR (11.4, IQR 7.9-18.3 beats per minute; 11.4/113, 10.1% error), noninvasive DBP (9.0, IQR 5.3-12.0 mm Hg; 9.0/58.6, 15.3% error), EtCO_2_ (4.0, IQR 2.0-5.8 mm Hg; 4.0/36.6, 10.9% error), and RR (2.0, IQR 0.4-4.2 breaths per minute; 2.0/20.4, 9.8% error), and the smallest median differences were in SpO_2_ (2.0%, IQR 1.4%-2.8%; 2.0/96, 2.1% error) and invasive SBP (4.0, IQR 3.5-7.6 mm Hg; 4.0/88.0, 4.5% error). Noninvasive SBP (8.3, IQR 5.0-13.5 mmHg; 8.3/99.4, 8.4% error), invasive DBP (3.8, IQR 3.0-5.0 mmHg; 3.8/50.0, 7.6% error rate) met concordance criteria. There were large outliers in most of the recorded vital signs. These included differences in HR of 87 beats per minute, RR of 18 breaths per minute, invasive SBP of 31 mm Hg, cuff DBP of 18 mm Hg, and cuff SBP of 21 mm Hg. Three variables failed to meet our concordance adequacy threshold of 10%: HR (11.4/113, 10.1%), cuff DBP (9/58.6, 15.3%), and EtCO_2_ (4.0/36.6, 10.9%).

**Table 2.** Differences in measured vital signs between the etiometry and electronic health record extracted measurements.

Measurements (n=110)	Difference, median (IQR)	Maximum value	Error rate, n (%)
Heart rate (beats per min)	11.4 (7.9-18.3)	87	11.4/113 (10.1)
Respiratory rate (breaths per min)	2.0 (0.4-4.2)	18	2.0/20.4 (9.8)
Systolic BP^a^ (mm Hg)—cuff	8.3 (5.0-13.5)	21	8.3/99.4 (8.4)
Diastolic BP (mm Hg)—cuff	9.0 (5.3-12.0)	18	9/58.6 (15.3)
Systolic BP (mm Hg)—invasive	4.0 (3.5-7.6)	31	4.0/88.0 (4.5)
Diastolic BP (mm Hg)—invasive	3.8 (3.0-5.0)	15	3.8/50.0 (7.6)
Oxygen saturation (%)	2.0 (1.4-2.8)	6	2.0/96 (2.1)
End-tidal carbon dioxide (mm Hg)	4.0 (2.0-5.8)	10	4.0/36.6 (10.9)

a BP: blood pressure.

### Ventilator Data

Seven variables were measured regarding ventilator settings, and differences were compared between the research team bedside collection and the data extracted from the EHR (Table 3). No data were collected from Etiometry for ventilator settings. The EHR data were highly concordant regarding ventilator settings, with median differences of 0.0 for each of the 6 continuous variables and a 95.5% (105/110) concordance regarding the ventilator mode. Differences were observed occasionally, and while most were small (positive end-expiratory pressure of 1 mm Hg, fraction of inspired oxygen of 0.1%, and tidal volume difference of 1 cm^3^), some were large (RR of 34 breaths per minute and inspiratory time of 0.3 seconds). All ventilator variables met our concordance adequacy threshold of 10%.

**Table 3.** Differences between the ventilator settings measured at bedside and those extracted from the electronic health record measured in medians per patient.

Measurements (n=110)	Difference, median (IQR)	Maximum value	Error rate (%)
Tidal volume (cm^3^)	0.0 (0.0-0.0)	1	0
Pressure control (mm Hg)	0.0 (0.0-0.0)	0	0
Respiratory rate (breaths per min)	0.0 (0.0-0.2)	3	0
PEEP^a^ (mm Hg)	0.0 (0.0-0.0)	1	0
FiO_2_^b^ (%)	0.0 (0.0-0.0)	0.1	0
I-time^c^ (s)	0.0 (0.0-0.0)	0.3	0

a PEEP: positive end-expiratory pressure.

b FiO_2_: fraction of inspired oxygen.

c I-time: inspiratory time.

### Medication Data

**Figure 1.**
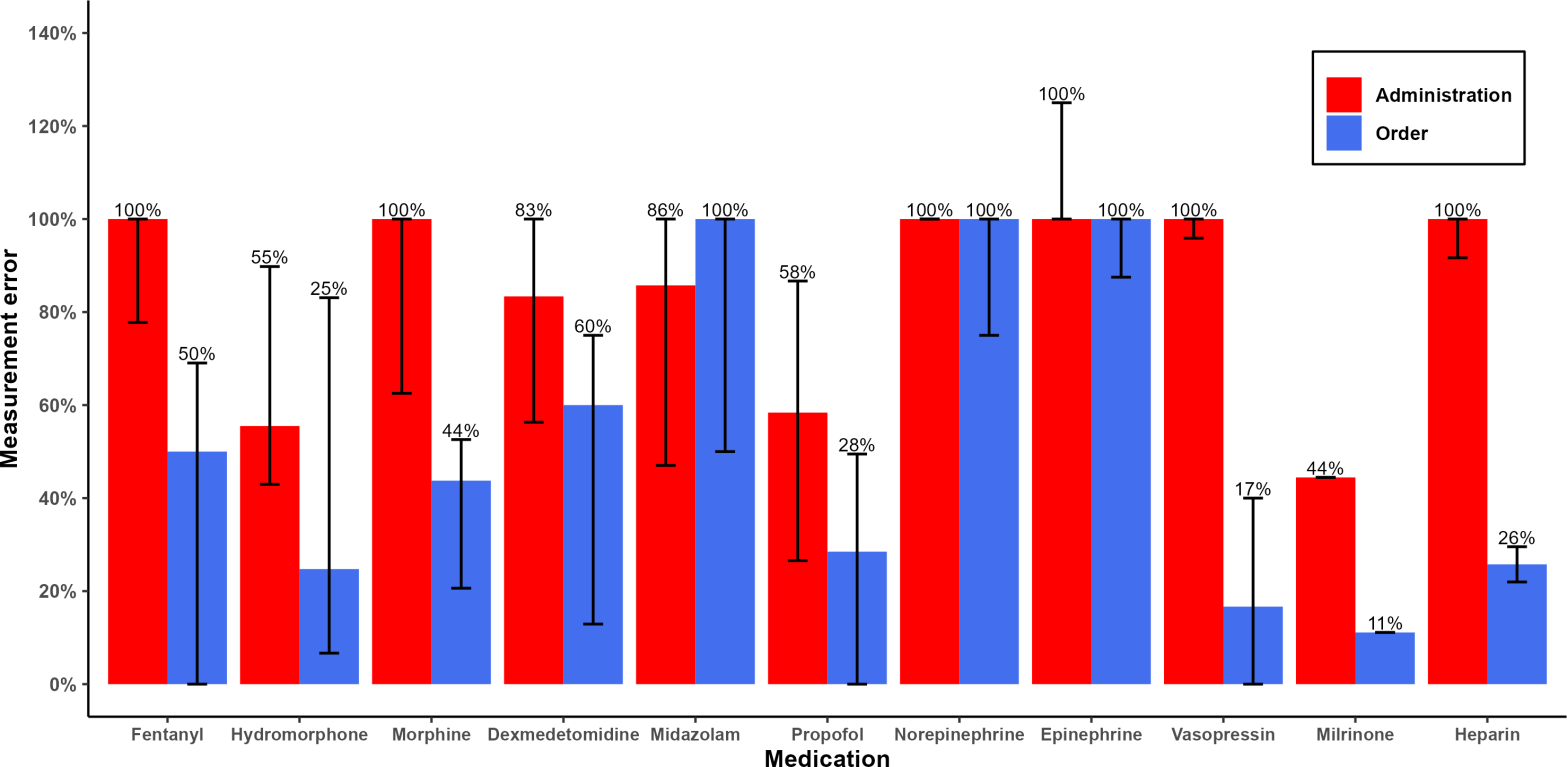
Grouped bar chart illustrating differences between medication infusion dosing collected by the bedside research team member and medication order and administration data obtained from the electronic health record. Differences are presented as medians with IQRs.

Dosing measurements from 11 continuously infusing medications were obtained from the EHR from 2 distinct tables (order and administration) and compared to the bedside recordings (Figure 1). The average patient-level concordance for the order table was 30.8% (SD 22.9%) between the bedside recordings and the EHR data. Of the 208 total observed errors, 111 (53.4%) were missing errors, and 97 (46.6%) were dose errors. The average patient-level concordance for the administration table was 31.4% (SD 15.5%) between the bedside recordings and the EHR data. Of the 199 total observed errors, 175 (87.9%) were missing errors, and 24 (12.1%) were dose errors. Regarding the order table, the medications with the largest percentage of error rates were midazolam (median 100%, IQR 50%-100%), norepinephrine (median 100%, IQR 75%-100%), and epinephrine (median 100%, IQR 88%-100%), and the medications with the smallest percentage of error rates were hydromorphone (median 25%, IQR 7%-83%), vasopressin (median 17%, IQR 0%-40%), propofol (median 29%, IQR 0%-50%), milrinone (median 11%, IQR 11%-11%), and heparin (median 0%, IQR 0%-9%; Table S3 in Multimedia Appendix 1). Regarding the administration table, the medications with the largest percentage of error rates were fentanyl (median 100%, IQR 78%-100%), morphine (median 100%, IQR 63%-100%), norepinephrine (median 100%, IQR 100%-100%), epinephrine (median 100%, IQR 100%-125%), vasopressin (median 100%, IQR 96%-100%), and heparin (median 100%, IQR 92%-100%), and the medications with the smallest percentage of error rates were hydromorphone (median 55%, IQR 43%-90%), propofol (median 58%, IQR 27%-87%), and milrinone (median 44%, IQR 44%-44%). Comparing the order table results to the administration table results, the order table had a lower overall concordance than the administration table, but the difference was not statistically significant (30.8% vs 31.4%; *P*=.33). In comparing the percentage of error, the order percentage of error was significantly lower than the administration percentage of error (median 50%, IQR 7%-92% vs median 100%, IQR 58%-100%; *P*<.001).

## Discussion

### Principal Findings

We compared the clinical data for a sample of pediatric patients on mechanical ventilators in the PICU obtained from 3 different sources. In our sample, we found that, for vital signs, data obtained from the EHR were concordant for 5 out of 8 vital signs with Etiometry data. For ventilator settings, we found that all measured variables in the EHR data were concordant with real-time bedside measurements. We also found that, for continuous medication infusions, both the order and administration tables obtained from the EHR were largely discordant.

Vital signs are monitored continuously for many hospitalized patients and for nearly all patients in the PICU. They are essential elements for clinical care in this population because meaningful changes in physiology can occur rapidly, and PICU clinicians must recognize and respond to these changes. These measures are valuable for clinical researchers as they can reflect periods of acute deterioration or illustrate responses to clinical interventions. Thus, their accuracy in readily available sources is important. Our study found that 5 of 8 measured vital signs recorded in the EHR were concordant with those obtained through the validated bedside monitoring system. On average, there were small to moderate differences observed across the recorded vital signs. However, we did observe rare large differences. While we were unable to confirm this in our study, these larger differences could be due to errors in data entry by the bedside staff. These errors could be keystroke errors or field entry errors unintentionally substituting the value of one vital sign for another [29]. This is a known weakness of EHR systems caused by human error but does add bias to clinical data captured in EHR systems. It is also possible that device artifacts or measurement errors caused these outliers. These results show that, in our sample, most extracted vital signs from EHR systems were concordant with continuous physiological monitoring data and can potentially be used for observational research. However, attention should be paid to outliers in the data, and methods to address the potential bias that these outliers add may be needed.

Ventilator settings are important for PICU clinicians to have available as they can provide valuable information regarding patients’ respiratory and neurological physiology. We found that, in our sample, all ventilator settings captured in the EHR met concordance criteria. This is likely due to the low frequency of changes in ventilator settings relative to other measured study elements. While practice varies widely between PICU physicians and institutions, ventilator settings are often changed only a few times per day if the patient is not having acute issues. The ability to extract accurate ventilator settings from the EHR enables clinical researchers to conduct studies evaluating ventilator management strategies and disease trajectories, and the finding that EHR-extracted ventilator elements were concordant with bedside measurements is valuable to these future research efforts.

Continuous medications are commonly used in the PICU for sedation, shock management, and other conditions [30,31]. The ability to accurately capture the dosing regimens for these medications is important to conduct robust research regarding standard sedation protocols, sepsis resuscitation practices, and other vital PICU practice areas. Our study showed that, while medication dosing was captured routinely in the EHR, the concordance of the dosing was highly variable. While this variability was not equal for all medications, the overall concordance for all medications was low. Clinically, this is an understandable finding, particularly in the PICU setting. Clinical scenarios change rapidly in the PICU, and one purpose of continuous infusions is the ability to adjust dosing rapidly depending on the patient’s situation. Rapid changes typically happen through verbal orders at the bedside, such as when an intubated patient is not adequately sedated and requires quick response to prevent endotracheal tube dislodgement or a patient with septic shock with poor hemodynamics who requires increased vasoactive medication to maintain adequate oxygen delivery. Often, these medication adjustments happen at the bedside, and then after a time delay, the orders are changed in the EHR. Regarding administration data, these are often scanned into the EHR at the time the medication infusion is started and then updated once the medication infusion runs out and needs to be renewed. These time delays between actual dosing change and updates in the EHR likely account for most of the discordance measured in our study. This is supported by the types of errors measured, with the order errors having a similar proportion of dose errors and missing errors, and the administration errors being primarily missing errors. Other possible reasons for the observed errors could be timing discrepancies or incorrect dose entry on the medication pumps compared to the desired medication dose in the EHR. It is possible that, as clinical informatics systems improve their capabilities through more direct measurement systems, such as Etiometry, this discordance will also improve and enhance our capacity to conduct robust observational research on continuous medication infusions.

Our study has several strengths and limitations. The strengths include bedside data collection by a trained clinical research team member and the use of the novel continuous vital sign monitoring system data. Limitations include a short time window of less than a month and a total of only 27 unique patients with multiple repeated observations at a single institution, which may add bias and limit generalizability. While most EHR-captured data points were within an expected distribution, there were several larger outliers. These may represent charting errors caused either by substitution of an HR value for an RR value or missed keystrokes that can lead to inaccurate data collection but could also be due to other causes. The time windows used for measurement matching were wide, which enhances usable matched data but may also introduce bias by matching observations inaccurately. In addition, bedside collection was only performed during daytime hours, and it is possible that concordance may differ during night hours. Bedside monitoring data may also contain inaccuracies such as artifacts and sensor displacement, which would bias our data. Our population was PICU patients, which have unique considerations in clinical care and data collection that may limit the application of our findings to patients in other areas of the hospital.

### Conclusions

We found that EHR data capture for intubated children in our single-center sample in the PICU was mostly concordant with data capture from continuous monitoring systems for vital signs, concordant with bedside observations for ventilator settings, and discordant with bedside measurement for continuous medications. Further studies with larger sample sizes from other institutions are needed to validate these findings.

## Supplementary material

10.2196/93017 Multimedia Appendix 1 Supplemental tables, STROBE checklist, and adjusted Bland-Altman plots for the vital sign and ventilator data.

## Abbreviations

DBP: diastolic blood pressure
EHR: electronic health record
EtCO_2_: end-tidal carbon dioxide
HR: heart rate
ICU: intensive care unit
PICU: pediatric intensive care unit
RR: respiratory rate
SBP: systolic blood pressure
SpO_2_: oxygen saturation
STROBE: Strengthening the Reporting of Observational Studies in Epidemiology
