# Supplementary material for: Assessment of Electronic Clinical Monitoring Systems in the Pediatric Intensive Care Unit: Prospective Concordance Study
Source: JMIR Form Res. 2026 Aug 11;10:e93017. doi: 10.2196/93017 (PMC13460674; doi:10.2196/93017)
Supplement: Supplementary file 1 [file formative-v10-e93017-s001.docx]

Supplementary Materials

1. Page 2: Supplemental Table 1
2. Page 3: Supplemental Table 2
3. Page 4: Supplemental Table 3
4. Pages 5-10: STROBE checklist
5. Page 11-: Supplemental Figure 1
6. Page 11-: Supplemental Figure 2
7. Page 12-: Supplemental Figure 3
8. Page 12-: Supplemental Figure 4
9. Page 13-: Supplemental Figure 5
10. Page 13-: Supplemental Figure 6
11. Page 14-: Supplemental Figure 7
12. Page 14-: Supplemental Figure 8
13. Page 15-: Supplemental Figure 9
14. Page 15-: Supplemental Figure 10
15. Page 16-: Supplemental Figure 11
16. Page 16-: Supplemental Figure 12
17. Page 17-: Supplemental Figure 13
18. Page 17-: Supplemental Figure 14

Supplemental Table 1: Data elements collected by each of the three methods.

| **Bedside Collection** | **Electronic Health Record** | **Etiometry** |
| --- | --- | --- |
| **Vital Signs** | **Vital Signs** | **Vital Signs** |
| Heart Rate (beats per minute) | Heart Rate (beats per minute) | Heart Rate (beats per minute) |
| Respiratory Rate (breaths per minute) | Respiratory Rate (breaths per minute) | Respiratory Rate (breaths per minute) |
| Systolic Blood Pressure (Cuff) (mmHg) | Systolic Blood Pressure (Cuff) (mmHg) | Systolic Blood Pressure (Cuff) (mmHg) |
| Diastolic Blood Pressure (Cuff) (mmHg) | Diastolic Blood Pressure (Cuff) (mmHg) | Diastolic Blood Pressure (Cuff) (mmHg) |
| Systolic Blood Pressure (Invasive) (mmHg) | Systolic Blood Pressure (Invasive) (mmHg) | Systolic Blood Pressure (Invasive) (mmHg) |
| Diastolic Blood Pressure (Invasive) (mmHg) | Diastolic Blood Pressure (Invasive) (mmHg) | Diastolic Blood Pressure (Invasive) (mmHg) |
| Oxygen Saturation (%) | Oxygen Saturation (%) | Oxygen Saturation (%) |
| End Tidal Carbon Dioxide (mmHg) | End Tidal Carbon Dioxide (mmHg) | End Tidal Carbon Dioxide (mmHg) |
| **Ventilator Settings** | **Ventilator Settings** |  |
| Mode | Mode |  |
| Pressure Control (mmHg) | Pressure Control (mmHg) |  |
| Tidal Volume (cc) | Tidal Volume (cc) |  |
| PEEP (mmHg) | PEEP (mmHg) |  |
| Fraction of Inspired Oxygen (%) | Fraction of Inspired Oxygen (%) |  |
| Inspiratory Time (sec) | Inspiratory Time (sec) |  |
| **Medication Infusions** | **Medication Infusions** |  |
| Morphine (mg/kg/hr) | Morphine (mg/kg/hr) |  |
| Hydromorphone (mcg/kg/hr) | Hydromorphone (mcg/kg/hr) |  |
| Fentanyl (mcg/kg/hr) | Fentanyl (mcg/kg/hr) |  |
| Midazolam (mg/kg/hr) | Midazolam (mg/kg/hr) |  |
| Dexmedetomidine (mcg/kg/hr) | Dexmedetomidine (mcg/kg/hr) |  |
| Propofol (mcg/kg/hr) | Propofol (mcg/kg/hr) |  |
| Epinephrine (mcg/kg/min) | Epinephrine (mcg/kg/min) |  |
| Norepinephrine (mcg/kg/min) | Norepinephrine (mcg/kg/min) |  |
| Vasopressin (milliunits/kg/min) | Vasopressin (milliunits/kg/min) |  |
| Dopamine (mcg/kg/min) | Dopamine (mcg/kg/min) |  |
| Milrinone (mcg/kg/min) | Milrinone (mcg/kg/min) |  |
| Heparin (u/kg/hr) | Heparin (u/kg/hr) |  |

Supplementary Table 2: Data Completeness Data

| **Data Element** | **Standard (n)** |  |
| --- | --- | --- |
|  | **Etiometry** | **EHR (n[%])** |
| Heart Rate | 110 | 110 (100) |
| Respiratory Rate | 110 | 110 (100) |
| Oxygen Saturation | 109 | 110 (100) |
| End Tidal CO2 | 109 | 109 (100) |
| SBP Cuff | 94 | 94 (100) |
| DBP Cuff | 94 | 94 (100) |
| SBP Arterial Line | 17 | 17 (100) |
| DBP Arterial Line | 17 | 17 (100) |
|  | **Bedside** |  |
| Ventilator Mode | 110 | 110 (100) |
| Ventilator Tidal Volume | 104 | 103 (99) |
| Ventilator Pressure Control | 6 | 2 (33) |
| Ventilator Rate | 110 | 108 (99) |
| Ventilator PEEP | 110 | 110 (100) |
| Ventilator FiO2 | 110 | 110 (100) |
| Inspiratory Time | 110 | 108 (98) |

CO2=Carbon Dioxide, SBP=Systolic Blood Pressure, DBP=Diastolic Blood Pressure, PEEP=Positive End Expiratory Pressure, FiO2=Fraction of Inspired Oxygen

Supplementary Table 3: Medications

| **Medication** | **Order Concordance**  **(%)** | **Admin Concordance**  **(%)** | **p-value** | **Order Error**  **(%)** | **Admin Error**  **(%)** | **p-value** |
| --- | --- | --- | --- | --- | --- | --- |
| Fentanyl | 38.5 | 23.1 | 0.42 | 50 (0-69) | 100 (78-100) | *0.01 |
| Hydromorphone | 33.3 | 41.7 | 0.73 | 25 (7-83) | 55 (43-90) | 0.63 |
| Morphine | 6.3 | 18.8 | 0.29 | 44 (21-53) | 100 (63-100) | *0.02 |
| Dexmedetomidine | 23.3 | 32.5 | 0.36 | 60 (13-75) | 83 (56-100) | *0.02 |
| Midazolam | 13.9 | 38.9 | 0.06 | 100 (50-100) | 86 (47-100) | 0.47 |
| Propofol | 56.2 | 31.2 | 0.26 | 29 (0-50) | 58 (27-87) | 0.26 |
| Norepinephrine | 13.3 | 38.9 | 0.34 | 100 (75-100) | 100 (100-100) | 0.76 |
| Epinephrine | 0.0 | 33.3 | 0.17 | 100 (88-100) | 100 (100-125) | 0.17 |
| Vasopressin | 62.5 | 0.0 | 0.07 | 17 (0-40) | 100 (96-100) | *0.03 |
| Milrinone | 66.7 | 33.3 | 1.0 | 11 (11-11) | 44 (44-44) | 1.0 |
| Heparin | 25.0 | 62.5 | 0.47 | 26 (22-30) | 100 (92-100) | 0.09 |
| Overall | 30.8 | 31.4 | 0.33 | 50 (7-92) | 100 (58-100) | *<0.01 |

Hypothesis testing done using Wilcoxon Rank Sum Tests

*Statistically significant at an alpha level of 0.05

STROBE Statement—checklist of items that should be included in reports of observational studies

|  | Item No. | Recommendation | Page  No. | Relevant text from manuscript |
| --- | --- | --- | --- | --- |
| **Title and abstract** | 1 | (*a*) Indicate the study’s design with a commonly used term in the title or the abstract | 1 | Assessment of Electronic Clinical Monitoring Systems  in the Pediatric Intensive Care Unit: A prospective cohort study |
|  |  | (*b*) Provide in the abstract an informative and balanced summary of what was done and what was found | 2 |  |
| Introduction | | | |  |
| Background/rationale | 2 | Explain the scientific background and rationale for the investigation being reported | 3 |  |
| Objectives | 3 | State specific objectives, including any prespecified hypotheses | 3 | We hypothesized that data captured in the EHR would be less accurate than data captured by the other stated methods. |
| Methods | | | |  |
| Study design | 4 | Present key elements of study design early in the paper | 3-6 | We conducted a prospective cohort study at a single, quaternary care, academic pediatric institution. |
| Setting | 5 | Describe the setting, locations, and relevant dates, including periods of recruitment, exposure, follow-up, and data collection | 4-5 | The study population included all patients admitted to the Riley Hospital PICU receiving invasive mechanical ventilation during the study period. Invasive ventilation included both via endotracheal tube or tracheostomy. We chose this population to maximize the data available for the study, as these patients will have regular data collected for vital signs, ventilator data, and continuous medications. Children at end of life or with significant social concerns that could be exacerbated by the research team member presence were excluded. The study period was June 4, 2025, to June 27, 2025. |
| Participants | 6 | (*a*) *Cohort study*—Give the eligibility criteria, and the sources and methods of selection of participants. Describe methods of follow-up  *Case-control study*—Give the eligibility criteria, and the sources and methods of case ascertainment and control selection. Give the rationale for the choice of cases and controls  *Cross-sectional study*—Give the eligibility criteria, and the sources and methods of selection of participants | 4-5 | The study population included all patients admitted to the Riley Hospital PICU receiving invasive mechanical ventilation during the study period. Invasive ventilation included both via endotracheal tube or tracheostomy. |
|  |  | (*b*) *Cohort study*—For matched studies, give matching criteria and number of exposed and unexposed  *Case-control study*—For matched studies, give matching criteria and the number of controls per case | NA |  |
| Variables | 7 | Clearly define all outcomes, exposures, predictors, potential confounders, and effect modifiers. Give diagnostic criteria, if applicable | 4-6 |  |
| Data sources/ measurement | 8* | For each variable of interest, give sources of data and details of methods of assessment (measurement). Describe comparability of assessment methods if there is more than one group | *3-5* |  |
| Bias | 9 | Describe any efforts to address potential sources of bias | 4-6 |  |
| Study size | 10 | Explain how the study size was arrived at | 3-4 |  |

Continued on next page

| Quantitative variables | 11 | Explain how quantitative variables were handled in the analyses. If applicable, describe which groupings were chosen and why | 4-6 |  |
| --- | --- | --- | --- | --- |
| Statistical methods | 12 | (*a*) Describe all statistical methods, including those used to control for confounding | 5-6 |  |
|  |  | (*b*) Describe any methods used to examine subgroups and interactions | NA |  |
|  |  | (*c*) Explain how missing data were addressed | 4-5 |  |
|  |  | (*d*) *Cohort study*—If applicable, explain how loss to follow-up was addressed  *Case-control study*—If applicable, explain how matching of cases and controls was addressed  *Cross-sectional study*—If applicable, describe analytical methods taking account of sampling strategy | NA |  |
|  |  | (*e*) Describe any sensitivity analyses | NA |  |
| Results | | | | |
| Participants | 13* | (a) Report numbers of individuals at each stage of study—eg numbers potentially eligible, examined for eligibility, confirmed eligible, included in the study, completing follow-up, and analysed | 6 | Over the 23-day study period, we conducted 18 bedside data collections on 27 unique patients, resulting in 110 total bedside observations. Patients had a median age of 6 years (IQR: 0.9-12.5) and a variety of primary diagnoses. |
|  |  | (b) Give reasons for non-participation at each stage | NA |  |
|  |  | (c) Consider use of a flow diagram | NA |  |
| Descriptive data | 14* | (a) Give characteristics of study participants (eg demographic, clinical, social) and information on exposures and potential confounders | 6 |  |
|  |  | (b) Indicate number of participants with missing data for each variable of interest | 6 |  |
|  |  | (c) *Cohort study*—Summarise follow-up time (eg, average and total amount) | 6 |  |
| Outcome data | 15* | *Cohort study*—Report numbers of outcome events or summary measures over time | *6-8* |  |
|  |  | *Case-control study—*Report numbers in each exposure category, or summary measures of exposure |  |  |
|  |  | *Cross-sectional study—*Report numbers of outcome events or summary measures |  |  |
| Main results | 16 | (*a*) Give unadjusted estimates and, if applicable, confounder-adjusted estimates and their precision (eg, 95% confidence interval). Make clear which confounders were adjusted for and why they were included | 6-8 |  |
|  |  | (*b*) Report category boundaries when continuous variables were categorized | 6-8 |  |
|  |  | (*c*) If relevant, consider translating estimates of relative risk into absolute risk for a meaningful time period | NA |  |

Continued on next page

| Other analyses | 17 | Report other analyses done—eg analyses of subgroups and interactions, and sensitivity analyses | NA |  |
| --- | --- | --- | --- | --- |
| Discussion | | | | |
| Key results | 18 | Summarise key results with reference to study objectives | 9-10 |  |
| Limitations | 19 | Discuss limitations of the study, taking into account sources of potential bias or imprecision. Discuss both direction and magnitude of any potential bias | 10 |  |
| Interpretation | 20 | Give a cautious overall interpretation of results considering objectives, limitations, multiplicity of analyses, results from similar studies, and other relevant evidence | 9-10 |  |
| Generalisability | 21 | Discuss the generalisability (external validity) of the study results | 9-10 |  |
| Other information | |  | | |
| Funding | 22 | Give the source of funding and the role of the funders for the present study and, if applicable, for the original study on which the present article is based | NA |  |

*Give information separately for cases and controls in case-control studies and, if applicable, for exposed and unexposed groups in cohort and cross-sectional studies.

**Note:** An Explanation and Elaboration article discusses each checklist item and gives methodological background and published examples of transparent reporting. The STROBE checklist is best used in conjunction with this article (freely available on the Web sites of PLoS Medicine at http://www.plosmedicine.org/, Annals of Internal Medicine at http://www.annals.org/, and Epidemiology at http://www.epidem.com/). Information on the STROBE Initiative is available at www.strobe-statement.org.

Supplemental Figure 1: Adjusted Bland-Altman plot showing mean absolute measurement differences in heart rate (beats per minute) per patient.

Supplemental Figure 2: Adjusted Bland-Altman plot showing mean absolute measurement differences in respiratory rate (breaths per minute) per patient.

Supplemental Figure 3: Adjusted Bland-Altman plot showing mean absolute measurement differences in non-invasive systolic blood pressure (millimeters of mercury) per patient.

Supplemental Figure 4: Adjusted Bland-Altman plot showing mean absolute measurement differences in non-invasive diastolic blood pressure (millimeters of mercury) per patient.

Supplemental Figure 5: Adjusted Bland-Altman plot showing mean absolute measurement differences in invasive systolic blood pressure (millimeters of mercury) per patient.

Supplemental Figure 6: Adjusted Bland-Altman plot showing mean absolute measurement differences in invasive diastolic blood pressure (millimeters of mercury) per patient.

Supplemental Figure 7: Adjusted Bland-Altman plot showing mean absolute measurement differences in oxygen saturation (percent) per patient.

Supplemental Figure 8: Adjusted Bland-Altman plot showing mean absolute measurement differences in end tidal carbon dioxide (millimeters of mercury) per patient.

Supplemental Figure 9: Adjusted Bland-Altman plot showing mean absolute measurement differences in ventilator tidal volume (cubic centimeters) per patient.

Supplemental Figure 10: Adjusted Bland-Altman plot showing mean absolute measurement differences in pressure control settings (millimeters of mercury) per patient.

Supplemental Figure 11: Adjusted Bland-Altman plot showing mean absolute measurement differences in ventilator respiratory rate (breaths per minute) per patient.

Supplemental Figure 12: Adjusted Bland-Altman plot showing mean absolute measurement differences in positive end expiratory pressure (millimeters of mercury) per patient.

Supplemental Figure 13: Adjusted Bland-Altman plot showing mean absolute measurement differences in the fraction of inspired oxygen (percent) per patient.

Supplemental Figure 14: Adjusted Bland-Altman plot showing mean absolute measurement differences in ventilator inspiratory time (seconds) per patient.
